# A Case of Slowly Progressive Type 1 Diabetes Mellitus (SPIDDM, Probable) Successfully Managed With Oral Semaglutide for Glycemic Control and Weight Management Accompanied by Changes in Eating Behavior

**DOI:** 10.7759/cureus.96335

**Published:** 2025-11-07

**Authors:** Taro Hirai, Munehiro Kitada, Keita Endo, Koichi Hayashi, Toshihiko Suzuki

**Affiliations:** 1 Nephrology, Endocrinology, and Diabetes, Tokyo Bay Urayasu Ichikawa Medical Center, Urayasu, JPN; 2 Internal Medicine, Hamada Medical Clinic, Wakayama, JPN; 3 Nephrology, Wakayama Medical University, Wakayama, JPN; 4 Department of Emergency and Critical Care Medicine, St. Marianna University School of Medicine, Kawasaki, JPN

**Keywords:** eating behavior, glucagon-like peptide-1 receptor agonists (glp-1 ras), glycemic control, latent autoimmune diabetes in adults (lada), oral semaglutide, slowly progressive type 1 diabetes mellitus (spiddm), weight management

## Abstract

The role of glucagon-like peptide-1 receptor agonists (GLP-1RAs) as a standard treatment for slowly progressive type 1 diabetes mellitus (SPIDDM), a condition akin to latent autoimmune diabetes in adults (LADA) or latent autoimmune diabetes in the young (LADY), and a term primarily used in Japan, remains to be fully established. However, these agents show potential as a therapeutic option. We report a case of a 23-year-old Japanese woman who had SPIDDM (probable) since the age of 19 and had poor glycemic and weight control despite treatment with insulin, dapagliflozin, and metformin. Following the initiation of oral semaglutide (a GLP-1 RA), significant improvements in glycemic levels and weight were observed, enabling the discontinuation of insulin. Notably, beneficial changes in her eating behavior were also evident after oral semaglutide administration. This case suggests that GLP-1 RAs are a beneficial therapeutic option for glucose and weight management in patients with SPIDDM (probable), particularly when accompanied by favorable alterations in eating behavior, thus warranting further investigation.

## Introduction

Type 1 diabetes mellitus (T1DM) typically involves autoimmune destruction of pancreatic β-cells, resulting in insulin deficiency and rapid progression to absolute insulin dependency [1], and is primarily distinguished from type 2 diabetes mellitus (T2DM), manifesting insulin resistance, relative insulin deficiency, and the absence of islet autoantibodies. Slowly progressive type 1 diabetes mellitus (SPIDDM) is a subtype of T1DM defined in Japan, and the disease entity is included in latent autoimmune diabetes in adults (LADA) or latent autoimmune diabetes in the young (LADY) [2]. SPIDDM is characterized by the presence of islet cell autoantibodies, including anti-glutamic acid decarboxylase antibody (anti-GAD Ab), leading to a gradual decline in β-cell function and eventual insulin dependence. At diagnosis, insulin secretory capacity is reportedly preserved [3] and early insulin administration reduces the rate of progression to insulin dependency compared to sulfonylurea (SU) in non-insulin-dependent patients with SPIDDM and a high anti-GAD Ab titer [4]. Standard therapeutic strategies for non-insulin-dependent patients with SPIDDM, however, remain to be established thus far.

Glucagon-like peptide-1 (GLP-1) is an incretin hormone that stimulates glucose-dependent insulin secretion and suppresses glucagon secretion. It also delays gastric emptying and has an appetite-suppressing effect, which can amplify the metabolic control [5]. The analogues of this hormone, the glucagon-like peptide-1 receptor agonists (GLP-1RAs), are widely used in the treatment of T2DM and obesity, and their beneficial role in cardiovascular effects has now been established [6]. By contrast, GLP-1 RAs are not an approved treatment for T1DM. A previous randomized controlled trial using liraglutide in T1DM showed mixed benefits, including glycemic and weight control, but was associated with an increased risk of hypoglycemia and ketosis [7]. Nevertheless, retrospective data suggest a potential benefit in specific T1DM subsets (e.g., those with obesity and preserved self-insulin secretion), such as SPIDDM, but robust trials are still needed to establish safety and efficacy [6].

Herein, we report a case of young-onset SPIDDM (probable) in which oral semaglutide, a GLP-1 RA, offers a favorable impact on glycemic control, weight management, and eating behavior. In this specific case, faced with the challenge of suboptimal glycemic control, persistent overweight, and strong reluctance toward injectable insulin, we utilized off-label oral semaglutide as a strategic therapeutic option.

## Case presentation

The patient was a 23-year-old Japanese woman who first presented with glycosuria at the age of 17. At 19 years old, glycosuria was again noted during a visit to a primary care physician. Her postprandial plasma glucose level was 285 mg/dL, and her HbA1c level was 10.1%. She was subsequently referred to a diabetologist for further evaluation and treatment. At the initial presentation, her body weight (BW) was 55.8 kg, and her body mass index (BMI) was 23.3 kg/m². Based on a fasting plasma glucose level (118 mg/dL), fasting serum C-peptide immunoreactivity (CPR, 1.5 ng/mL), and a 24-hour urinary CPR (36.8 μg/day), definite insulin deficiency was ruled out. Although the test for urinary ketone bodies was positive (1+), her blood gas pH (7.398) and HCO3− (22.3 mmol/L) levels were within the normal range. Eventually, anti-GAD Ab was detected (802 U/mL, measured by the enzyme immunoassay (EIA) method). Hence, the patient was diagnosed with non-insulin-dependent SPIDDM; her clinical course and the absence of a family history of DM also ruled out maturity-onset diabetes of the young.

She was initially treated with insulin (insulin degludec/insulin aspart) once daily, along with dapagliflozin 5 mg/day, to attempt to achieve glycemic targets. The dapagliflozin dose was subsequently increased to 10 mg/day with careful monitoring for urinary ketones, and intensive insulin therapy was initiated. Furthermore, metformin was started at 500 mg/day and escalated to 1,500 mg/day owing to persistently elevated glucose levels. Despite these comprehensive therapies, optimal glycemic control was not achieved, and her BMI increased to a maximum of 28.8 kg/m² during this period.

 At 22 years of age, the patient was referred to our hospital due to relocation. Her initial clinical and laboratory data upon referral are summarized in Table 1.

**Table 1:** Initial laboratory data at our hospital

Laboratory test	Results	Reference value
Urine		
Protein	±	Negative
Glucose	4+	Negative
Ketone	2+	Negative
Cell blood counts		
White blood cell (WBC)	6600 /μl	4000-8000 /μl
Red blood cell (RBC)	660 × 10^4^ /μL	380-480 × 10^4^ /μl
Hemoglobin (Hb)	16 g/dL	12-16 g/dL
Platelet (Plt)	26.6 × 10^4^ /μl	15-35 × 10^4^ /μl
Biochemistry		
Sodium (Na)	139 mEq/L	138-146 mEq/L
Potassium (K)	4.1 mEq/L	3.6-4.9 mEq/L
Chloride (Cl)	101 mEq/L	99-109 mEq/L
Blood urea nitrogen (BUN)	19 mg/dL	8-22 mg/dL
Creatinine (Cr)	0.52 mg/dL	0.47-0.79 mg/dL
Estimated glomerular filtration rate (eGFR)	121 ml/min/1.73m^2^	>60 ml/min/1.73m^2^
Total protein (TP)	8.5 g/dL	6.7-8.3 g/dL
Albumin (Alb)	4.9 g/dL	3.1-5.1 g/dL
Aspartate aminotransferase (AST)	20 U/L	13-33 U/L
Alanine aminotransferase (ALT)	36 U/L	8-42 U/L
Gamma-glutamyl transpeptidase (GGTP)	18 U/L	10-47 U/L
Glucose metabolism		
Fasting plasma glucose (FPG)	99 mg/dL	70-109 mg/dL
C-peptide immunoreactivity (CPR)	0.87 ng/mL	1.1-3.3 ng/mL
Hemoglobin A1c (HbA1c)	8.5%	4.6-6.2%
Anti-glutamic acid decarboxylase antibody (anti-GAD Ab)	492.5 U/mL	0-4.99 U/mL
Anti-insulinoma-associated protein 2 antibody (anti-IA-2 Ab)	26.8 U/mL	0-0.59 U/mL
Thyroid-related data		
Thyroid-stimulating hormone (TSH)	0.986 μIU/mL	0.35-4.94 μIU/mL
Free thyroxine 4 (FT4)	1.06 ng/dL	0.70-1.72 ng/dL
Free thyroxine 3 (FT3)	2.77 pg/mL	1.88-3.86 pg/mL
Anti-thyroid peroxidase antibody (anti-TPO Ab)	<3.0 IU/mL	<3.0 IU/mL
Anti-thyroglobulin antibody (anti-Tg Ab)	18.4 IU/mL	<5.0 IU/mL

**Figure 1:**
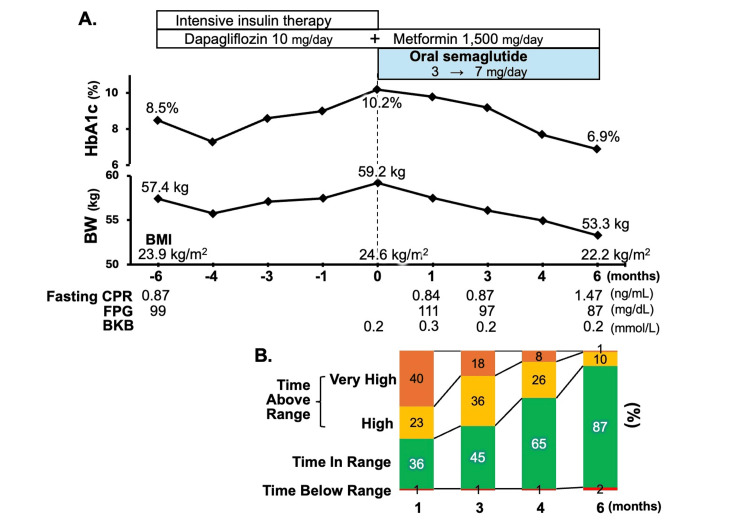
Clinical and glycemic parameters before and after oral semaglutide administration. Clinical course before and after oral semaglutide administration (A). The hemoglobin A1c (HbA1c) level improved from 10.2% to 6.9% following six months of oral semaglutide administration. Body weight (BW) decreased from 59.2 kg to 53.3 kg, and the body mass index (BMI) declined from 24.6 kg/m² to 22.2 kg/m² during the same timeframe. Change in time in range (TIR) measured by intermittently scanned continuous glucose monitoring (isCGM) after oral administration of semaglutide (B). The rate of TIR improved from 36% to 87% following six months of oral semaglutide administration. HbA1c: hemoglobin A1c; BMI: body mass index; BW: body weight; CPR: C-peptide immunoreactivity; FPG: fasting plasma glucose; BKB: blood ketone bodies This figure was created by the authors using Microsoft Excel and Microsoft PowerPoint (Microsoft Corp., WA, USA).

Her HbA1c level was 8.5%, BMI was 23.9 kg/m², and fasting CPR level was 0.87 ng/mL. Urinary ketone bodies showed positive (2+) during fasting while taking dapagliflozin; however, she did not exhibit clinical symptoms such as significant thirst, polyuria, polydipsia, or malaise. Her anti-GAD Ab and anti-insulinoma-associated protein 2 (IA-2) antibody were double positive. She was on dapagliflozin (10 mg/day) and metformin (1,500 mg/day) (Figure 1A). In addition, intensive insulin therapy with insulin aspart (six to eight units three times daily before meals) and insulin degludec (16 units once daily) was prescribed. However, her adherence to self-injecting insulin and monitoring of blood glucose levels was poor, primarily due to difficulty accepting self-injection, leading to frequent skipped injections multiple times per week. Adherence to nutritional and exercise therapy was also inadequate. Consequently, her HbA1c level and BMI increased to 10.2% and 24.6 kg/m², respectively.

**Figure 2:**
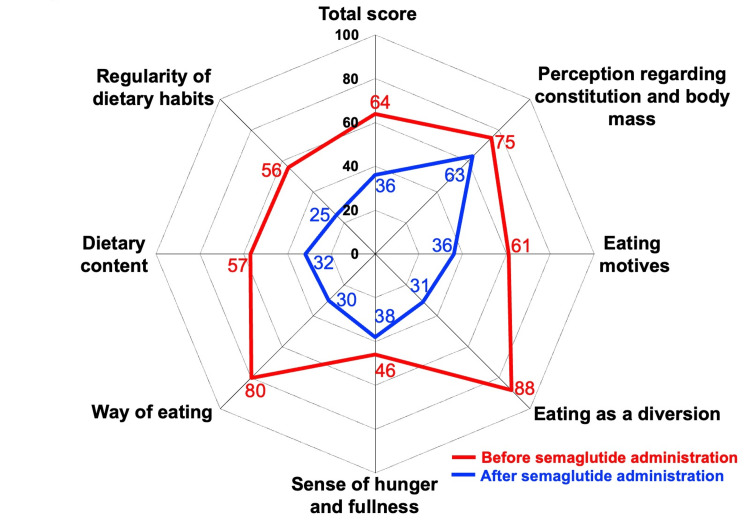
Changes in eating behavior profile with oral semaglutide administration. This radar chart illustrates scores for various aspects of eating behavior. Eating behavior was quantified using the 55-item dietary behavior questionnaire developed by the Japan Society for the Study of Obesity (JASSO) [8,9], which spans seven domains: regularity of dietary habits, dietary content, way of eating, sense of hunger and fullness, eating as a diversion, eating motives, and perception regarding constitution and body mass. Each item was rated on a four-point scale ranging from 1 (seldom) to 4 (very often). Higher scores in each domain indicate a greater deviation or stronger undesirable tendency in that particular eating habit. The red and blue lines represent scores before and after oral semaglutide administration, respectively.

Considering these challenges, oral semaglutide was introduced at 3 mg/day, replacing insulin therapy to address her persistent metabolic difficulties (Figure 1A). The dose was then titrated to 7 mg/day, resulting in improved glycemic control and no evidence of ketosis within one month of insulin cessation. Objective glycemic data, as assessed by intermittently scanned continuous glucose monitoring (isCGM), revealed a remarkable improvement in time in range (TIR) from one to six months post-initiation of oral semaglutide (Figure 1B). Oral semaglutide was well-tolerated throughout the six-month treatment period, with no adverse events reported that required dose adjustment or drug discontinuation. Furthermore, obvious alterations in her eating behaviors, including slower eating and a decreased frequency of snacking, were observed. As illustrated in Figure 2, these eating behaviors were quantified using the 55-item dietary behavior questionnaire developed by the Japan Society for the Study of Obesity (JASSO) [8,9]

In this questionnaire, each item was rated on a four-point scale from 1 (seldom) to 4 (very often), and higher scores for each domain indicate a greater deviation or stronger undesirable tendency in eating behavior. These favorable changes paralleled the reductions in both BMI (to 22.2 kg/m²) and HbA1c (to 6.9%) over six months. Concurrently, her fasting serum CPR level was maintained (1.47 ng/mL).

## Discussion

Herein, we report a case of non-insulin-dependent young-onset SPIDDM where oral semaglutide led to successful glycemic control and BW management and remarkable improvement in eating habits. Recently, the Japan Diabetes Society proposed new diagnostic criteria for SPIDDM. Conventional non-insulin-dependent SPIDDM is classified as SPIDDM (probable) based on (1) the presence of islet-associated autoantibodies, (2) the absence of ketoacidosis and unnecessary use of insulin treatment, and (3) the absence of severe endogenous insulin deficiency (serum CPR < 0.6 ng/mL) [2]. Although our patient received insulin therapy to alleviate hyperglycemia at the time of diagnosis, ketoacidosis was not observed. Furthermore, preserved insulin secretory capacity (CPR: 0.87 ng/mL) was maintained for more than three months (Figure 1). Based on these findings, we diagnosed this case with SPIDDM (probable).

This case provides valuable insights into the role of GLP-1 RAs in the management of SPIDDM, particularly when conventional therapies fall short. Our patient initially struggled with inadequate glycemic and BW control despite a comprehensive regimen including insulin, dapagliflozin, and metformin. The subsequent administration of oral semaglutide resulted in remarkable improvements in glycemic parameters, which enabled insulin discontinuation and a substantial reduction in BW (Figure 1A). These favorable outcomes were notably accompanied by beneficial changes in her eating behaviors (Figure 2). This observed dietary pattern aligns with the approach suggested by the JASSO [8]. Similar efficacy of semaglutide in glycemic and BW management, often associated with alterations in eating behavior, has been reported in Japanese obese type 2 diabetic patients [10]. Consistent with these findings, therefore, a beneficial trend is anticipated in SPIDDM patients.

Of note, the use of SGLT2 inhibitors carries a well-documented risk of ketoacidosis. This recognized risk has resulted in a divergent international regulatory landscape. Specifically, the Food and Drug Administration (FDA) has not approved the use of SGLT2 inhibitors for type 1 diabetes. By contrast, the European Medicines Agency (EMA) has approved low-dose dapagliflozin (5 mg) and sotagliflozin (200 mg) for patients with a BMI ≥ 27 kg/m² [11]. In addition, conditional use is permitted exclusively for dapagliflozin and ipragliflozin in Japan. In our case, dapagliflozin was initially selected due to preserved C-peptide function (SPIDDM probable) and the need for effective weight management. To mitigate the risk of ketoacidosis, our therapeutic protocol included initiating treatment at a low dose (5 mg/day) and implementing monitoring of serum ketones. Thus, our deliberate, risk-mitigating approach underpinned the clinical decision-making process for using this agent in the T1DM subtype.

The observed improvements in glycemic control and BW can be attributed to both the pancreatic and extrapancreatic effects of GLP-1 RAs. The beneficial changes in the patient's eating behaviors were particularly noteworthy and are likely to play a crucial role in her BW reduction and improved glycemic control. GLP-1 RAs are known to modulate eating behaviors through various central and peripheral mechanisms, including enhanced satiety, suppressed appetite and hunger, reduced preference for energy-dense foods, and alterations in food-reward pathways [12-14]. Supporting these mechanisms, liraglutide, another GLP-1 RA, was demonstrated to suppress central nervous system activation in response to food cues in obese type 2 diabetic patients [15]. This finding suggests that GLP-1RAs directly influence the brain's reward system and appetite regulation centers, leading to suppressed food cravings and improved eating control observed in our patient. The specific changes in her eating patterns, such as slower eating and a decreased frequency of snacking, directly align with these known effects, suggesting that an improved eating pattern substantially contributes to the successful outcome by reducing caloric intake and improving metabolic efficiency. The patient's initial suboptimal glycemic control and gradual BW gain were likely exacerbated by lifestyle-related factors, including poor adherence to dietary and exercise regimens and a tendency toward being overweight, akin to observations in T2DM. Furthermore, insulin monotherapy without accompanying lifestyle modifications may have contributed to BW gain in this patient. Given that effective BW management is crucial in patients with both T1DM and T2DM, GLP-1 RAs are anticipated to be of particular benefit in treating T1DM in patients with obesity, including those with SPIDDM, due to their favorable extra pancreatic effects [16].

In this case, oral semaglutide was selected over an injectable GLP-1 RA due to the patient's difficulty accepting self-injection of insulin. In contrast, adherence to oral medications, including semaglutide, was well maintained. Generally, adherence to injectable regimens tends to be poorer than that to oral medications, and many patients are hesitant regarding injections despite the critical importance of glycemic control [17]. Oral medications may therefore help alleviate the reluctance to administer injectable treatments.

The Japan Diabetes Society recently proposed a clinical statement regarding the treatment of SPIDDM (probable) [18]. Early intervention with insulin therapy reportedly slows the progression to insulin dependence in non-insulin-dependent SPIDDM compared to the administration of SU agents [4,18]. However, not all SPIDDM patients require early initiation of insulin therapy [18]. In our case, insulin therapy was switched to oral semaglutide, which resulted in favorable glycemic control and maintained levels of CPR over the 6-month treatment (CPR: 0.87-1.47 ng/mL, Figure 1A). GLP-1 promotes the proliferation and survival of β-cells, decreases apoptosis in experimental models of T1DM [5,19]. In addition, in patients with T1DM, GLP-1 RAs, such as liraglutide, improve residual β-cell function and reduce the insulin dosage during the first year following diagnosis [20]. Although the sustained CPR levels are consistent with improved metabolic control (e.g., reduced glucotoxicity), it requires further investigations to clarify whether GLP-1RAs also directly contribute to β-cell protection in patients with SPIDDM.

Finally, in this case, insulin therapy was withdrawn after the oral administration of semaglutide. Nevertheless, careful monitoring of insulin secretion is warranted because high anti-GAD Ab titers are associated with an increased risk of progression to β-cell dysfunction [3].

## Conclusions

Our findings suggest that GLP-1RAs, including semaglutide, serve as a therapeutic option for patients with SPIDDM (probable), particularly overweight patients. This approach effectively achieved glycemic targets and favorably managed BW. These benefits are likely to be attributable in part to the observed beneficial alterations in eating behavior.
